# Current and future perspectives for structural biology at the Grenoble EPN campus: a comprehensive overview

**DOI:** 10.1107/S1600577525002012

**Published:** 2025-04-14

**Authors:** Andrew A. McCarthy, Shibom Basu, Florent Bernaudat, Matthew P. Blakeley, Matthew W. Bowler, Philippe Carpentier, Gregory Effantin, Sylvain Engilberge, David Flot, Frank Gabel, Lukas Gajdos, Jos J. A. G. Kamps, Eaazhisai Kandiah, Romain Linares, Anne Martel, Igor Melnikov, Estelle Mossou, Christoph Mueller-Dieckmann, Max Nanao, Didier Nurizzo, Petra Pernot, Alexander Popov, Antoine Royant, Daniele de Sanctis, Guy Schoehn, Romain Talon, Mark D. Tully, Montserrat Soler-Lopez

**Affiliations:** a European Molecular Biology Laboratory (EMBL) Grenoble France; b European Synchrotron Radiation Facility (ESRF) Grenoble France; c Institut Laue–Langevin (ILL) Grenoble France; d Univ. Grenoble Alpes, CNRS, CEA, IRIG–LCBM UMR 5249, Grenoble, France; e Univ. Grenoble Alpes, CNRS, CEA, Institut de Biologie Structurale (IBS), Grenoble, France; Cornell University, USA

**Keywords:** structural biology, Grenoble EPN campus, fourth-generation synchrotrons, macromolecular crystallography

## Abstract

The European Photon and Neutron campus in Grenoble is a unique site, encompassing the European Synchrotron Radiation Facility Extremely Brilliant Source, the Institut Laue–Langevin, the European Molecular Biology Laboratory and the Institut de Biologie Structurale. Here, we present an overview of the structural biology beamlines, instruments and support facilities available on the EPN campus. These include advanced macromolecular crystallography using neutrons or X-rays, small-angle X-ray or neutron scattering, cryogenic electron microscopy, and spectroscopy. These highly complementary experimental approaches support cutting-edge research for integrated structural biology in our large user community. This article emphasizes our significant contributions to the field, outlines current advancements made and provides insights into our future prospects, offering readers a comprehensive understanding of the EPN campus’s role in advancing integrated structural biology research.

## Abstract 2

Details on the current status of the experimental techniques available at the ESRF–EBS and ILL research infrastructures are provided. Major contributions made by the EMBL, ESRF, ILL and IBS to the structural biology field are described, including future prospects at the EPN campus

## 1. Introduction

**Figure 1:**
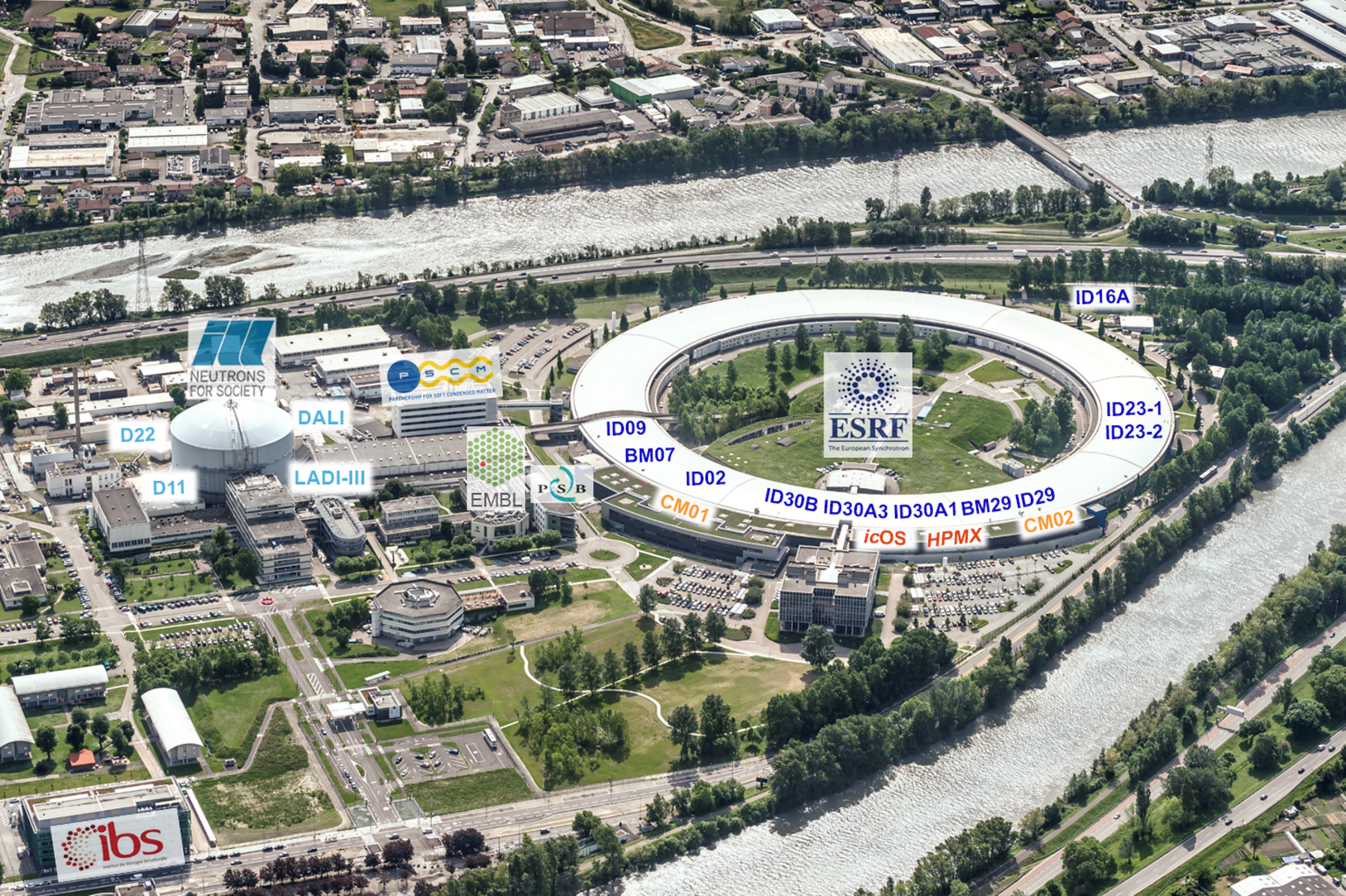
Aerial view of the Grenoble EPN campus, highlighting the PSB partners: EMBL, ESRF, IBS and ILL. ILL instruments and ESRF beamlines and facilities are indicated. The ILL D-Lab and the ESRF Structural Biology laboratory are in the CIBB building as part of the PSB partnership. Photograph courtesy of Denis Morel.

The European Photon and Neutron (EPN) Science Campus is unique in hosting three intergovernmental scientific research organizations and a French research institute (Fig. 1 ▸). It all started in 1967 when the Institute Laue–Langevin (ILL), the world’s most intense neutron source, was founded and the first neutrons delivered in 1971. A few years later, in 1975, the European Molecular Biology Laboratory (EMBL) established an outstation (now unit) adjacent to the ILL to study biological problems using neutrons. This led to many fruitful collaborations with a joint ILL/EMBL development of the first neutron image-plate diffractometer, LADI, a major technical achievement that increased the efficiency of neutron diffraction experiments (Cipriani *et al.*, 1995 ▸). Subsequent upgrades have further extended the size and complexity of the systems now studied using neutron diffraction on LADI-III (Blakeley *et al.*, 2010 ▸). Around the 1980s the EMBL Grenoble started to strengthen its methodological approaches to studying macromolecules using electron microscopy and X-rays. This effort was given a major boost in 1989 when the site underwent a major expansion following establishment of the European Synchrotron Radiation Facility (ESRF), now the world’s first fourth-generation high-energy X-ray source (Reichert, 2019 ▸). To further leverage the scientific potential of the ESRF and ILL for structural biology, the ‘Commissariat à l’Énergie Atomique et aux Énergies Alternatives’ (CEA) and the ‘Centre National de la Recherche Scientifique’ (CNRS) also founded the ‘Institut de Biologie Structurale’ (IBS) in 1992, with the ‘Université Joseph Fourier’ (now part of Université Grenoble Alpes) joining in 1999, and eventually moving onto the EPN campus in 2013.

Pre-1990, the majority of macromolecular crystallography (MX) structures were determined using laboratory-based X-ray sources. This started to change dramatically in the 1990s with the development of the multi-wavelength anomalous diffraction (MAD) method for the experimental determination of macromolecular structures, for which synchrotron radiation was essential (Hendrickson, 1999 ▸). The ESRF was the world’s first third-generation synchrotron when it commenced user operation in 1994 with a small complement of beamlines. These undulator-based sources were initially shared for MX experiments (Helliwell & Mitchell, 2015 ▸) and a dedicated bending magnet source on BM14 soon followed in 1995 (Cassetta *et al.*, 1999 ▸). The ESRF and EMBL Grenoble played a leading role in these early MX developments, with the first successful MAD structure determination of a macromolecule, ytterbium N-cadherin, from an undulator source (ID10) reported in 1995 (Shapiro *et al.*, 1995 ▸). The small highly collimated X-ray beams from undulator sources were also shown to be invaluable for small weakly diffracting membrane and virus crystals, as exemplified by the structure determination of bacteriorhodopsin to 2.5 Å (Pebay-Peyroula *et al.*, 1997 ▸) and bluetongue virus core to 3.5 Å (Grimes *et al.*, 1998 ▸) on ID13 and ID02, respectively. These early efforts led to the establishment of the ESRF–EMBL joint structural biology group (JSBG), a long and fruitful collaboration aimed at co-development of state-of-the-art user facilities at the ESRF, which has been in existence since 1996. A major early technical achievement from this collaboration was the first-generation, highly innovative micro-diffractometer (MD1), which enabled user-friendly diffraction measurements from micrometre-sized crystals on the ID13 microfocus beamline (Perrakis *et al.*, 1999 ▸).

These ‘pioneering era (1994–2000)’ successes supported efforts to expand available beam time on undulator sources, starting with the innovative Quadriga beamline complex dedicated to MX on ID14 (Wakatsuki *et al.*, 1998 ▸). Early experiments on this suite of beamlines showed the importance of crystal cryopreservation in helping mitigate against radiation damage (Garman, 2003 ▸). Further studies showed that this effect had to be carefully considered during data collection experiments on undulator sources because it induced chemical changes and degraded experimental data (Ravelli & McSweeney, 2000 ▸). These effects could be monitored by spectroscopy methods (McGeehan *et al.*, 2009 ▸; McGeehan *et al.*, 2007 ▸) and modelled to statistically estimate the optimal diffraction dose parameters to be used (Bourenkov & Popov, 2010 ▸). The ID14 complex was closely followed by ID29 in 2001 (de Sanctis *et al.*, 2012 ▸), which coincided with the transfer of BM14 to the UK Medical Research Council (MRC) to be collaboratively run with EMBL Grenoble. In 2002, the Partnership for Structural Biology (PSB) in Grenoble (https://www.psb-grenoble.eu/) was established, encompassing the EMBL, ESRF, IBS and ILL. In 2006, the PSB inaugurated the Carl-Ivar Brändén Building (CIBB) that was initially shared with the ‘Institut de Virologie Moléculaire et Structurale’ (IVMS, associated with the UJF and CNRS) to house the Centre for Integrated Structural Biology (CISB). In 2007, the IVMS evolved into the Unit for Viral Host Cell Interactions with the involvement of EMBL Grenoble and eventually, in 2016, the French groups of the unit were subsumed by IBS. This collaborative effort provided access to other technologies needed for protein crystallography pipelines, such as recombinant protein production and high-throughput crystallization (HTX), with EMBL Grenoble establishing an HTX platform within the PSB in 2003. The aims of this partnership were to establish a unique multi-disciplinary environment for integrated structural biology, where each institute would benefit from each other’s expertise and resources, and to create a European Centre of Excellence for the study of challenging biological problems, notably in human health.

In 2004 the tuneable ID23-1 beamline (Nurizzo *et al.*, 2006 ▸) commenced user operation and was soon complemented by the world’s first microfocus beamline dedicated to MX on ID23-2 in 2005 (Flot *et al.*, 2010 ▸), inspired by the pioneering experiments carried out on ID13. The innovative canted undulator configuration on ID23 was part of the ESRF’s contribution to the PSB. Though several other factors such as cryopreservation, recombinant protein production and HTX platforms were essential for the maturation of MX in Grenoble, instrument technology developments always played a critical role. These included the development by EMBL of the ultra-high precision MD2 diffractometer (later followed by the MD3). This was successfully commercialized by MAATEL/ARINAX (Moirans, France) under an EMBL licence and subsequently installed on many synchrotron sources worldwide. Another was the first generation of robotic sample changers, SC3, developed by EMBL, ESRF and BM14, accommodating up to 50 samples in 5 baskets under cryogenic conditions (Cipriani *et al.*, 2006 ▸). Lastly, reliable large-area CCD-based X-ray detectors from MAR Research and ADSC also became commercially available, facilitating accurate and faster collection of diffraction data. It was therefore clear early on that care, standardization and automation developments were required to efficiently use valuable MX beam time so that optimal experimental diffraction data could be collected. These included standard SPINE sample holders, robotic sample changers, smart software pipelines (including crystal realignment options with a mini-kappa goniometer head), a graphical user interface control (*MXCuBE*) and an Information System for the Protein crystallography Beamlines (*ISPyB*) experimental database (Flot *et al.*, 2006 ▸; Beteva *et al.*, 2006 ▸; Cipriani *et al.*, 2006 ▸; Incardona *et al.*, 2009 ▸; Brockhauser *et al.*, 2012 ▸; Gabadinho *et al.*, 2010 ▸; Delagenière *et al.*, 2011 ▸) that were all co-developed for the 7 undulator-based ESRF–EMBL JSBG beamlines and then the UK Medical Research Council/EMBL operated BM14, with crucial support from many EU projects (SPINE, SPINE2-COMPLEXES and BIOXHIT). All these ‘automation era (2000–2010)’ efforts led to many seminal studies such as bacterial ribosome structures (Schlünzen *et al.*, 2001 ▸; Selmer *et al.*, 2006 ▸) and G-protein coupled receptor structures (Warne *et al.*, 2008 ▸) that were recognized with Nobel prizes in Chemistry in 2009 and 2012, respectively, to name a few.

Following a first successful decade of user operation, the ESRF published its future ‘Purple book’ vision in 2007 to provide a resource that would satisfy ESRF user needs for the next 10–15 years. Phase I of this ESRF upgrade program started in 2009 when eleven candidate upgrade beamlines were selected. In this ‘golden era (2010–2017)’ of MX it was clear that structural biologists were tackling ever more ambitious projects and that higher capacity robotic sample changers would enable users to screen larger numbers of variable quality crystals before selecting the best one(s) for their diffraction experiments. This led to the proposal (UBPL10) for a ‘massively automated sample selection integrated facility (MASSIF) for macromolecular crystallography’ to replace the ageing ID14 Quadriga complex with a modern suite of three new MX beamlines using a canted undulator source on ID30 (Bowler *et al.*, 2015 ▸; McCarthy *et al.*, 2018 ▸; von Stetten *et al.*, 2020 ▸). In addition, a highly automated small-angle X-ray scattering (SAXS) beamline dedicated to biological samples was built on BM29 (Pernot *et al.*, 2013 ▸; Round *et al.*, 2015 ▸). When complete in 2015, a total of six modern MX beamlines, including significant upgrades to PILATUS pixel array detectors (DECTRIS, Baden, Switzerland) (Broennimann *et al.*, 2006 ▸) and new EMBL–ESRF developed robotic sample changers on ID23-1, ID23-2 and ID29, were operated by the JSBG with complementary properties (automatic, microfocus and tuneable) (Mueller-Dieckmann *et al.*, 2015 ▸). Significant upgrades to the software stack were also carried out at this time, notably *MXCuBE2*, which integrated a *Beamline Expert System* server to facilitate complex experimental workflows (Oscarsson *et al.*, 2019 ▸). Additional MX beam time was also available from the Collaborative Research Group (CRG) beamlines on BM14 (run by an EMBL/India/ESRF consortium from 2010 until 2016) and BM30A-FIP (a French beamline operated by IBS from 1999 to 2018). Following the rapid growth of cryogenic electron microscopy (cryo-EM), the undulator source beamlines were complemented in 2017 by a Titan Krios microscope (FEI), CM01, managed by the ESRF and operated with EMBL and IBS partners (Kandiah *et al.*, 2019 ▸). Meanwhile, other long-established ancillary support facilities evolved and remained available to users, such as the *in crystallo* Optical Spectroscopy (*ic*OS) (von Stetten *et al.*, 2015 ▸) and high-pressure freezing for macromolecular crystallography (HPMX) (Carpentier *et al.*, 2024 ▸). Lastly, the EPN campus also enables neutron and X-ray diffraction or scattering experiments that provide complementary information to be carried out during a single visit (Drago *et al.*, 2024 ▸; Kim *et al.*, 2016 ▸), with a joint application system to combine small-angle neutron scattering (SANS) experiments at ILL with bioSAXS measurements at ESRF available since 2011.

The ESRF’s Upgrade Phase II, as envisaged in the ‘Orange book’, included a complete upgrade of the accelerator to a fourth-generation X-ray source, which was approved in 2014. Following careful planning and an 18 month shutdown starting in December 2018, the ESRF Extremely Brilliant Source (EBS) came into existence and user operation recommenced in August 2020 as foreseen (Raimondi *et al.*, 2023 ▸). In order to maximize the scientific potential of the exceptional ESRF–EBS X-ray source, several flagship beamline proposals were chosen. In structural biology, EBSL8, a new time-resolved serial synchrotron crystallography (TR-SSX) beamline on ID29 dedicated to the study of dynamic biological process down to a time resolution of 20 µs was selected (de Sanctis *et al.*, 2012 ▸; Orlans *et al.*, 2025 ▸). To remain competitive and maximize their scientific potential, all the ESRF structural biology beamlines underwent refurbishment.

Below we describe the current portfolio of neutron, X-ray and cryo-EM facilities available for academic and industrial structural biology users on the EPN campus and some future directions in this new ‘post cryo-EM/*AlphaFold* era’.

## 2. Current state of the structural biology facilities

Currently, the ESRF–EMBL JSBG offers a wide range of synchrotron beamlines, including five for MX [ID23-1, ID23-2, ID30A-1 (MASSIF-1), ID30A-3 (MASSIF-3) and ID30B], one for TR-SSX (ID29) and one for bioSAXS (BM29). Complementing these are two state-of-the-art cryo-EM facilities: CM01, managed by the ESRF and operated with EMBL and IBS; and CM02, a French CRG facility managed and operated by the IBS. The French CRG beamline BM07 (FIP2), which began operation in 2022, represents another significant addition. Furthermore, the ESRF has three dedicated facilities (a biochemistry laboratory, HPMX and *ic*OS) further enhancing structural biology capacities, while the EMBL Grenoble also provides limited HTX access services through externally funded mechanisms (Fig. 1 ▸, Table 1 ▸).

Additionally, the ILL supports biological neutron studies with a dedicated laboratory for deuteration of samples (D-Lab), two solution scattering SANS (D11 and D22) and two neutron crystallography (DALI and LADI-III) instruments, complementing the synchrotron-based X-ray crystallography offerings (Table 2 ▸).

### 2.1. Automation in the structural biology beamlines

Automation is pivotal to the EPN campus’s X-ray experimental workflows. As part of a recent upgrade, the software stack was fully refactored to adhere to modern, robust and maintainable open-source programming standards. Key updates included deploying the low-level BeamLine Instrumentation Support Software (BLISS) standard control system (Guijarro *et al.*, 2023 ▸) and high-level *MXCuBE-Web*, the latest-generation GUI from the *MXCuBE* collaborative project (Oscarsson *et al.*, 2019 ▸) that supports remote access experiments, and *BSXCuBE-Web* on BM29 (Tully *et al.*, 2023 ▸). *MXCuBE-Web* allows researchers to conduct automated experiments with ease through advanced workflow routines (Oscarsson *et al.*, 2019 ▸). These tools are integrated through the *MXCuBE-Web* interfaces, linking beamline control hardware and software through a modular *Extensible Workflow System* (*EWOKS*) for automated routines such as X-ray centring and *MXPress* pipelines (Svensson *et al.*, 2015 ▸). The process of experimental design and data handling is further streamlined by automated data-processing pipelines, which use the *ISPyB* database for cataloguing results (Brockhauser *et al.*, 2012 ▸; Delagenière *et al.*, 2011 ▸; Monaco *et al.*, 2013 ▸). Concurrently, the *ISPyB* experimental database has undergone continuous improvements and is now transitioning to the more flexible *ISPyB-DRAC*, which supports parallel data processing across multiple pipelines, including *XDSAPP*, *EDNAProc*, *GRENADES*, *xia2* and *autoPROC* (Vonrhein *et al.*, 2011 ▸; Sparta *et al.*, 2016 ▸; Monaco *et al.*, 2013 ▸).

### 2.2. Fully automated beamline: ID30A-1 (MASSIF-1)

MASSIF-1 (ID30A-1) has led the way in automating MX experiments (Bowler *et al.*, 2015 ▸; Svensson *et al.*, 2015 ▸; Svensson *et al.*, 2018 ▸) and was the world’s first beamline to implement workflows that fully automated the entire process required for MX experiments at synchrotrons (Svensson *et al.*, 2019 ▸; Bowler *et al.*, 2016 ▸). Its mode of operation (access via a flexible beam time booking system, payment of sample shipment costs to/from the home laboratory) has provided a new paradigm for beamline access at the ESRF. This focus on automation has enabled the development of workflows that offer unique new features such as unattended crystal harvesting and data collection. Continuous software advancements have been matched by significant hardware investments from both ESRF and EMBL. In the summer of 2020, the beamline underwent a significant upgrade. It transitioned from a custom-built RoboDiff diffractometer (ESRF) (Nurizzo *et al.*, 2016 ▸) to the standardized MD2S ultra-high-precision micro-diffractometer (EMBL) and flexible and high-throughput sample changer (FlexHCD) system (EMBL/ESRF) (Papp *et al.*, 2017 ▸). A year later, a crystal-harvesting robot developed by the EMBL HTX and Instrumentation teams (Felisaz *et al.*, 2019 ▸; Zander *et al.*, 2016 ▸; Cornaciu *et al.*, 2021 ▸) was also installed as part of the JSBG collaboration. The MASSIF-1 operation model, particularly its automated queuing system, has set the foundation for automation at the ESRF, providing a blueprint that has been extended to other MX beamlines to support diverse scientific requirements in both industry and academia, particularly for fragment screening campaigns that require high-resolution data.

### 2.3. Energy-tuneable beamlines: ID23-1, ID30B and BM07 (FIP2)

The ID23-1 and ID30B beamlines are designed for flexible energy tuning, supporting high-flux, high-resolution experimental techniques such as multi-wavelength anomalous diffraction (MAD) and absorption edge scanning. Both beamlines feature a high degree of automation, enabling in-person operation, remote or fully automated modes, with the latter two modes being the most commonly used. The ability to rapidly adjust the energy within the 6–20 keV range facilitates X-ray absorption edge studies for biologically important metals, as well as anomalous scattering from lighter elements like P and S, and experimental phasing options when required. Additionally, both can be equipped with a mini-kappa goniometer head for multi-orientation data collection, enhancing their experimental options (Brockhauser *et al.*, 2013 ▸). ID23-1 underwent a major refurbishment in 2021, when an EIGER2 X 16M CdTe sensor detector and an MD2S micro-diffractometer were installed, and the FlexHCD robotic sample changer was updated. This investment enabled complex experimental protocols, such as multi-dimensional scans for sample alignment that facilitated high-throughput data collection, with over 21 samples being routinely processed per hour. This makes ID23-1 particularly suitable for large-scale ligand binding studies, ensuring precise data collection from small crystals while its tunability allows easy identification of metal ions in biological samples.

ID30B (McCarthy *et al.*, 2018 ▸) has also undergone significant upgrades, including the installation of an EIGER2 X 9M detector and the replacement of the elliptical horizontal focusing mirror with 2D Be compound refractive lenses (CRLs). These improvements result in a minimal beam size of approximately 20 µm × 30 µm (vertical × horizontal) at the sample position, which can be expanded if necessary. The beamline currently provides a photon flux exceeding 10^1^^3^ photons s^−1^ at 14 keV. ID30B also features a plate gripper for *in situ* plate and microfluidic screening and data collection (Gavira *et al.*, 2020 ▸). Furthermore, an HC-Lab humidifier (Sanchez-Weatherby *et al.*, 2009 ▸) can also be set up for both room-temperature or controlled-dehydration experiments. Additionally, ID30B can be equipped with a Raman spectroscopy setup, enabling the online collection of spectra before and/or after diffraction experiments (von Stetten *et al.*, 2017 ▸). These spectra are typically recorded in a time- or dose-dependent manner, allowing for the identification of specific changes in protein vibrational modes while ensuring that most complex modes remain unchanged. This capability is further enhanced through close collaboration with the *ic*OS Lab. Additionally, ID30B has strong ties to the HPMX laboratory, enabling the collection of diffraction data from gas-derivatized crystals. Its tunability also allows for the collection of anomalous scattering data from crystals derivatized with noble gases such as Kr or Xe (Carpentier *et al.*, 2024 ▸).

The French CRG Beamline FIP2 has been operational since October 2021, succeeding BM30A-FIP (1999–2018) (Roth *et al.*, 2002 ▸). Its tuneable monochromatic beam (6–20 keV) offers a large, adjustable top-hat profile (200 µm × 100 µm to 250 µm × 250 µm, horizontal × vertical) with a moderate flux (∼6 × 10^1^^1^ photons s^−1^), optimized for low-dose MX and radiation damage studies at room temperature (Garman & Weik, 2023 ▸). Equipped with a FlexHCD sample changer (Papp *et al.*, 2017 ▸), it facilitates high-throughput cryogenic MX. It uniquely combines *in crystallo* UV–Vis absorption, fluorescence emission spectroscopy and X-ray diffraction using an online microspectrophotometer (McGeehan *et al.*, 2009 ▸), allowing real-time analysis of redox states affected by radiation damage (Bolton *et al.*, 2024 ▸), ultimately enabling optimization of X-ray data collection parameters so as to preserve the integrity of X-ray sensitive chemical groups, especially during data collections performed at room temperature. These features are particularly suited for large crystals (>50 µm × 50 µm × 50 µm) and enable light activation of crystals at both cryogenic and room temperatures, using the HC-Lab humidifier (Sanchez-Weatherby *et al.*, 2009 ▸). FIP2 also complements neutron diffraction of large crystals at ILL’s LADI-III and DALI instruments, making it an ideal choice for advanced structural studies.

### 2.4. Microfocus and serial crystallography beamlines: ID30A-3 (MASSIF-3), ID23-2 and ID29

ID30A-3 is a fixed-energy (12.81 keV), high-intensity mini-focus beamline (15 µm^2^ FWHM) optimized for medium-sized crystals (≥20 µm) (von Stetten *et al.*, 2020 ▸). Located on the first ID30 port, it shares a canted undulator source with ID30A-1, ensuring a highly intense beam that is focused vertically by white beam CRLs and horizontally by a bent multilayer mirror. The beamline is equipped with a high-speed EIGERX 4M detector capable of up to 750 frames per second, an MD2 micro-diffractometer and a FlexHCD sample changer (Papp *et al.*, 2017 ▸), supporting rapid and complex data collection strategies, including multi-crystal and TR-SSX experiments (Aumonier *et al.*, 2020 ▸).

ID23-2 is a dedicated microfocus beamline (Flot *et al.*, 2010 ▸) that has undergone two major upgrades, enhancing almost every component (Nanao *et al.*, 2022 ▸). Though the most recent upgrade predates the ESRF–EBS, all optical layout choices were designed with EBS integration in mind. The beamline now features a dual set of CRLs and multilayer mirrors, achieving a highly focused microbeam (>10^1^^3^ photons s^−1^) with a minimum size of 1.5 µm × 3 µm (FWHM). Its sample environment includes a FlexHCD sample changer (Papp *et al.*, 2017 ▸), an MD3Up high-precision multi-axis diffractometer and an EIGER2 X 9M detector, enabling versatile experiments that go beyond microcrystals to various sample types and conditions. The small, high-flux beam of ID23-2 supports a range of experiments, including synchrotron serial crystallography (SSX), diffractive mapping and raster scans of multiple crystal regions (Totir *et al.*, 2012 ▸). A plate gripper is also available on ID23-2 for *in situ* serial crystallography (iSX) data collection experiments and has been integrated into *MXCuBE-Web* to facilitate the structure determination of challenging drug targets at room temperature (Foos *et al.*, 2024 ▸).

ID29 is a pioneering beamline dedicated to room-temperature macromolecular SSX experiments (de Sanctis, 2021 ▸; Stubbs *et al.*, 2023 ▸; Malla *et al.*, 2025 ▸; Orlans *et al.*, 2025 ▸). It is optimized for time-resolved structural studies, providing high-resolution data at room temperature and complementing the work at microfocus beamlines and XFELs. ID29 provides a highly focused and intense (∼2 × 10^15^ photons s^−1^) pulsed X-ray beam to collect data from true microcrystals, enabling the study of time-dependent conformational changes at exposure times as brief as ∼10 µs. By supporting ligand-binding and fragment-screening studies with microcrystals, ID29 enables physiologically relevant data collection without the need for cryoprotection. Multiple sample delivery methods, including high-viscosity injectors, fixed targets (foils or chips) and tape drive systems, cater to a variety of experimental needs, from fast mixing processes to light-activated studies. Operating in sync with the EBS, ID29 features a dual-chopper system to create X-ray pulses, supporting efficient data acquisition while optimizing sample use. Its unique design serves as a model for high-throughput SSX beamlines at other fourth-generation synchrotrons (Malla *et al.*, 2025 ▸; Orlans *et al.*, 2025 ▸). ID29 also includes a secondary experimental hutch to support future non-standard and high-energy SSX experiments.

### 2.5. Small-angle X-ray scattering beamline

BM29 is a state of the art, high-throughput, BioSAXS specific beamline with two vacuum compatible sample exposure units, allowing standard measurements through a capillary (from 277 to 333 K) or a more flexible setup, the SEU2B, specialized for microfluidic devices (Tully *et al.*, 2023 ▸). The BioSAXS liquid handling robot (ARINAX) is temperature controlled (from 277 to 313 K) and can measure up to 384 samples per day in batch mode, while the integrated Shimadzu HPLC can measure 72 samples in size-exclusion chromatography (SEC)–SAXS. A new PILATUS3 2M detector is housed under vacuum to reduce background scattering and increase signal-to-noise levels, facilitating the measurement of macromolecules at lower concentrations (Tully *et al.*, 2023 ▸). To streamline sample collection, we have also designed and introduced a new intuitive control software, *BSXCuBE3* (Oskarsson *et al.*, 2019 ▸). Sample environments are fully integrated and new users and experts can set up and run samples with ease. In addition, an open source, automated data reduction and processing pipeline, *FreeSAS*, was developed that integrates with both *ISPyB* and *ISPyB-DRAC* (Kieffer *et al.*, 2022 ▸). Small-angle scattering on the EPN campus is well integrated. Any successful SANS proposal at the ILL will receive corresponding SAXS beam time on BM29, if required. This enables the same samples to be measured with both neutrons and X-rays.

### 2.6. Cryo-electron microscopy facilities: CM01 and CM02

**Figure 2:**
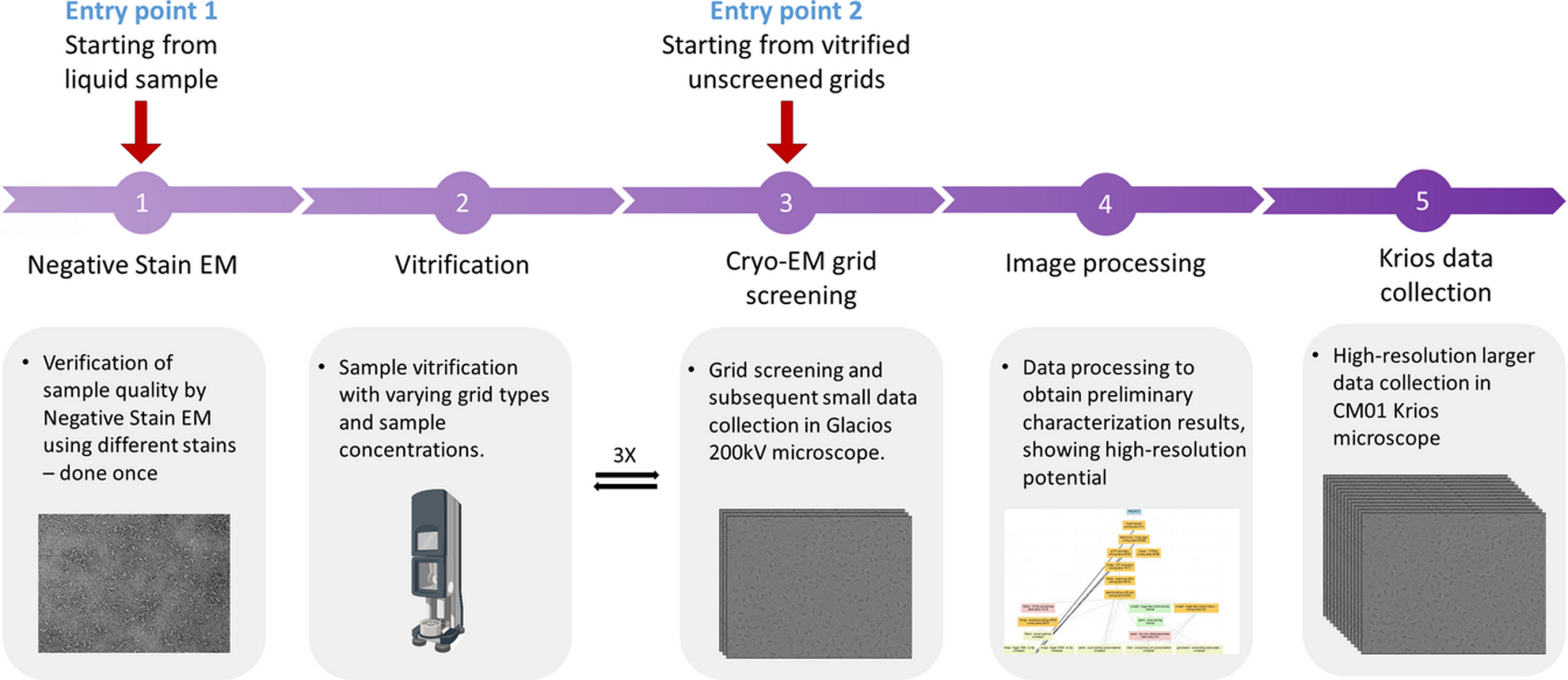
Workflow of the SOS pipeline for ESRF users at CM01: the pipeline, designed for users without regular access to a vitrification platform or screening microscope, follows the above structured approach at CM01, the cryo-EM beamline at ESRF. The workflow consists of five steps for liquid samples, beginning with negative-stain EM as a quality control step, and three steps for vitrified grids, starting directly with grid screening. Successful negative-stain EM leads to vitrification trials, followed by iterative cryo-EM grid screening to determine suitability for Krios data collection. Step 4 is currently performed by the user.

CM01 is equipped with cutting-edge technologies, including a Titan Krios G3 (TFS), a K3 direct electron detector (Gatan) coupled to a Quantum LS energy filter, complemented by a Volta phase plate (Kandiah *et al.*, 2019 ▸). CM01 supports both single-particle analysis (SPA) and cryo-electron tomography (cryo-ET), enabling high-resolution structural studies. SPA data collection is facilitated through the *EPU* software, achieving a throughput of 600–700 movies per hour. For cryo-ET, the *TOMO5* software is employed (Comet *et al.*, 2024 ▸). Successful SPA beam time allocation requires users to provide pre-characterized grids with optimal particle distribution and ice quality, along with high-resolution 2D class averages. A resolution of 1.62 Å for horse spleen apoferritin by SPA and 2.9 Å for the same protein via sub-tomogram averaging can be achieved. To facilitate user feedback, a real-time image-processing pipeline ‘*cryoEMProcess*’ that is based on the *SCIPION* framework has been implemented (Gómez-Blanco *et al.*, 2018 ▸; Maluenda *et al.*, 2019 ▸), which runs through to *ab initio* 2D classification, with results displayed in *ISPyB* (and soon *ISPyB-DRAC*). In collaboration with the IBS, the ESRF also offers a Solution-tO-Structure (SOS) pipeline, accommodating users starting from liquid protein samples or unscreened vitrified grids (Fig. 2 ▸) (Mueller-Dieckmann *et al.*, 2024 ▸). A new microscope, CM02, a French CRG, equipped with a Titan Krios G4 (cold-FEG), Falcon4i detector, Selectris X energy filter as well as a Fringe-Free Imaging (FFI) and Volta phase plate, further enhances the cryo-EM capabilities available to ESRF users.

### 2.7. Specialized ESRF laboratories and facilities

The *in crystallo* optical spectroscopy laboratory (*ic*OS Lab) provides offline microspectrophotometry measurements in proximity to the MX beamlines to gather spectroscopic data on crystals, complementing structural analysis. The main *ic*OS setup (von Stetten *et al.*, 2015 ▸) features an MD2M mini-diffractometer with three reflective objectives for UV–Vis absorption, fluorescence emission and a microscope objective for Raman spectroscopy, compatible with cryogenic and room temperatures using an HC-Lab humidity controller (Sanchez-Weatherby *et al.*, 2009 ▸). Time-resolved UV–Vis spectra can be obtained on the microsecond to second timescale using a pump–probe scheme on the TR-*ic*OS instrument (Engilberge *et al.*, 2024 ▸), supporting ID29 diffraction experiments. Online Raman spectroscopy is also available on ID30B, and UV–Vis absorption and fluorescence on FIP2 (McGeehan *et al.*, 2009 ▸). The *ic*OS Lab’s main applications include comparing protein crystal spectra to solution signatures to interpret crystallographic data, monitoring metal and cofactor signatures to assess X-ray radiation damage, and analysing reaction intermediates accumulated in crystals.

The high-pressure freezing laboratory for macromolecular crystallography (HPMX) specializes in studying gas–macromolecule interactions within crystals using gas-pressurizing cells (Lafumat *et al.*, 2016 ▸; Carpentier *et al.*, 2024 ▸). Originally, high-pressure techniques reduced cryoprotectant use or created noble gas derivatives (Barstow *et al.*, 2008 ▸; Schiltz *et al.*, 2003 ▸), but current research focuses on direct gas–macromolecule interactions. HPMX provides various cryogenic high-pressure cells for the cryo-trapping of gas–protein complexes using He, Ar, Kr, Xe, N_2_, O_2_, CO_2_ or CH_4_. This enables detailed mapping of functional protein architectures, including diffusion channels (Kalms *et al.*, 2016 ▸). Pressurizing gas-dependent enzyme crystals with gases such as N_2_, O_2_, CO_2_ or CH_4_ facilitates the elucidation of catalytic and gas transport mechanisms by trapping intermediates (Labidi *et al.*, 2023 ▸; Vilela-Alves *et al.*, 2024 ▸). Furthermore, high-pressure cryo-cooling at 2000 bar with He allows the observation of *in crystallo* protein conformational changes (Prangé *et al.*, 2022 ▸). With fewer than 1% of Protein Data Bank (PDB) structures featuring gas complexes, there is significant potential for advancing the study of protein–gas interactions. ID30B, equipped with online Raman spectroscopy and anomalous scattering capabilities, is well suited for locating gas molecules in high-pressure cryo-trapped crystals, as it can identify gases like Ar and Kr through anomalous difference maps.

The ESRF Structural Biology Group Laboratory at the PSB supports a growing demand for cross-disciplinary approaches in biology and biomedicine. This laboratory serves to train young researchers and to conduct pioneering research that inspires ESRF users while offering advanced technical support. It features dedicated spaces for molecular biology, biological sample production and crystallization, alongside newly implemented techniques for detecting protein–protein interactions in eukaryotic systems and performing cell biology assays. These advancements have yielded promising scientific outcomes, fostering new collaborations across ESRF facilities. This collaborative potential not only benefits the research projects of ESRF’s external users but also significantly enhances the research capabilities of ESRF scientists.

### 2.8. Neutron applications

Neutron scattering applications provide critical and unique insights into the structure and dynamics of biological systems at different time- and length-scales that are complementary to many other techniques, including X-ray scattering. Their unique advantages are maximized by the use of deuterium labelling. The ability to produce customized deuterium-labelled biological molecules enables the optimization of neutron studies in solution scattering, crystallography, reflectometry and dynamics, significantly enhancing the scope, quality and efficiency of research in these fields. Deuteration facilities have been established at many neutron centres worldwide, playing an important role in advancing neutron studies in the life sciences and soft matter research. The ILL is strongly committed to not only building high-performance instruments but also offering the best possible scientific environment for its user community.

#### 2.8.1. The ILL deuteration laboratory (D-Lab)

Established in the early 2000s and managed by the Biology, Deuteration, Chemistry and Soft Matter (BDCS) group since 2023, the D-Lab leverages deuterium (^2^H or D) labelling to enhance the sensitivity of neutron scattering to hydrogen isotopes. Different deuteration techniques can be applied based on specific neutron applications: neutron macromolecular crystallography (NMX) uses perdeuterated macromolecules or ligands to increase the signal-to-noise ratio, allowing for precise observation of H and D in single crystals (Drago *et al.*, 2024 ▸; Ramos *et al.*, 2021 ▸; Ramos *et al.*, 2025 ▸). NMX comprises about 20% of D-Lab projects; SANS uses variable deuteration levels to achieve contrast variation, facilitating studies of biomolecular complexes like protein–DNA or protein–membrane structures (Galvagnion *et al.*, 2024 ▸). SANS proposals make up around 50% of D-Lab’s work; neutron reflectometry (NR) benefits from maximum contrast between hydrogenated and deuterated molecules, useful for examining protein interactions with model membranes (Santamaria *et al.*, 2022 ▸); and neutron spectroscopy (NS), capitalizing on hydrogen’s incoherent cross-section, uses specific hydrogen labelling within deuterated samples to study molecular dynamics in complex systems like hydrated powders or cells (Wood *et al.*, 2013 ▸). The D-Lab has pioneered large-scale deuterated protein production through high-cell-density cultures (Haertlein *et al.*, 2016 ▸) and continues to refine *in vivo* deuteration techniques. Microorganisms such as bacteria and yeast are adapted to deuterated media to produce various labelled biomolecules (proteins, protein–nucleic acid complexes, glycoproteins and lipid nanodiscs), supporting advanced structural biology studies.

The recently established Lipid Lab (L-Lab), which has now been integrated into the BDCS group, has enabled users to produce more realistic biomimetic model membrane systems (containing the full range of biological lipids) for neutron scattering experiments. To achieve this, the L-Lab has been focusing on optimizing methods for the extraction and purification of H and D small molecules, including diverse lipid mixtures from microorganisms. Such model membranes show great promise toward a better understanding of the structure and function of biological membranes as well as their mechanisms of interaction with biomolecules such as proteins and drugs (Corucci *et al.*, 2023 ▸).

#### 2.8.2. Neutron solution scattering: D11 and D22

D11 and D22 are the primary small-angle neutron biological solution scattering (SANS) instruments at ILL. D22 offers an optimal flux-to-noise ratio and a combined SAXS/SANS setup (Metwalli *et al.*, 2021 ▸), whereas D11 enables access to smaller *Q*-values (Matsarskaia *et al.* 2023 ▸). Both instruments feature a velocity selector, segmental collimation and a tube detector, accommodating a wide range of sample environments due to their large sample zones. D22 has recently integrated a SEC–SANS for BioSANS experiments (Martel *et al.*, 2023 ▸) and added a second detector for full*Q*-range curves (0.003–0.6 Å^−1^) in one exposure. This setup, along with a semi-transparent beamstop for simultaneous transmission and scattering recording, is advantageous for SEC–SANS and time-resolved studies. BioSANS excels in studying the internal structures of bio-macromolecular complexes, with contrast variation (H_2_O–D_2_O exchange) and deuteration enhancing analysis of complex protein interactions (Yunoki *et al.*, 2022 ▸; Lapinaite *et al.*, 2020 ▸; Lycksell *et al.*, 2021 ▸; Maric *et al.*, 2014 ▸; Sonntag *et al.*, 2017 ▸). Supported by complementary methods like BioSAXS, NMR and cryo-EM, BioSANS can track molecular processes over extended periods due to the absence of radiation damage (Dicko *et al.*, 2020 ▸; Mahieu *et al.*, 2020 ▸). A partnership between ILL SANS and ESRF BioSAXS on the EPN campus allows users to benefit from integrated BioSAXS and BioSANS measurements. The PSB and the ESRF-ILL Partnership for Soft Condensed Matter (PSCM) biophysics platforms further support sample preparation and characterization (https://pscm-grenoble.eu/).

#### 2.8.3. Neutron crystallography: LADI-III, DALI

NMX complements MX by accurately locating H and D atoms in macromolecular structures at 1.5–2.5 Å resolution. Due to lower fluxes, larger crystals and longer collection times are required when compared with X-rays, crystal volumes are typically in the range ∼0.1 to 1.0 mm^3^ (depending on unit-cell volume, extent of deuteration, crystal mosaicity *etc*.). This technique, using non-destructive neutron wavelengths (>2.5 Å), allows data collection from a single crystal at room- or cryo-temperatures (Kwon *et al.*, 2016 ▸) without radiation damage. Identifying H/D positions provides crucial insights into protonation, hydrogen bonding and hydration, aiding enzymatic studies (Drago *et al.*, 2024 ▸), ligand-binding analysis (Gajdos *et al.*, 2022 ▸; Yee *et al.*, 2019 ▸) and structure-based drug design (Kneller *et al.*, 2022 ▸). The D-Lab supports crystal growth and mounting, and offers perdeuteration to reduce crystal volumes and improves signal quality. For enhanced data collection efficiency, the Laue method is combined with a large cylindrical detector at both diffractometers, LADI-III and DALI. LADI-III, operational since 2012, uses a multilayer filter for wavelength selection (typically ∼2.8 ≤ λ ≤ 3.8 Å) and it is optimized for unit-cell dimensions up to 80 Å. LADI-III holds a leading position for neutron structures in the PDB, while using many of the smallest crystals (Gajdos *et al.*, 2022 ▸; Yee *et al.*, 2019 ▸). DALI, introduced in 2021 for the study of larger unit-cell systems, features a velocity selector, providing higher transmission and narrower bandwidth, enhancing data quality with reduced reflection overlap.

## 3. Future developments and perspectives

The future of structural biology at the EPN campus in Grenoble is ready to embrace a new ‘post cryo-EM/*AlphaFold* era’ of multiscale and multimodal studies by integrating our cutting-edge technologies to address complex biological questions. To date, *AlphaFold2* and *AlphaFold3* (*AF2*, *AF3*) predictions are based on static structures, most of which were obtained at cryogenic temperatures, reflecting the methods deployed to obtain structural information. Therefore, to fundamentally understand how macromolecules function we must now probe their dynamic properties, as these are crucial to their function. By combining the strengths of X-rays, electron microscopy and neutron-based techniques, the EPN campus aims to provide access to the critical scientific research infrastructure needed by our user community to study biological systems at multiple temporal resolutions using integrated approaches. Strategic developments will focus on key areas such as room-temperature data collection, including fragment/ligand screening capabilities, as well as the ability to perturb and study biological systems in a time resolved manner. We believe the implementation of automation capabilities are crucial to broaden the number of systems that can be studied. The complementary use of SAXS/SANS for solution-state studies and the advanced spectroscopy methods available at *ic*OS will also provide valuable insights on macromolecular dynamics. However, biology also occurs in context, therefore breakthroughs in cryo-EM, cryo-ET and X-ray tomography will provide detailed structural and spatial information, bridging the gap between molecular and cellular biology. Together, these innovations will solidify the EPN campus as a hub for integrated structural biology, driving forward interdisciplinary research with transformative impacts on life sciences.

### 3.1. Future developments in automation and high throughput

**Figure 3:**
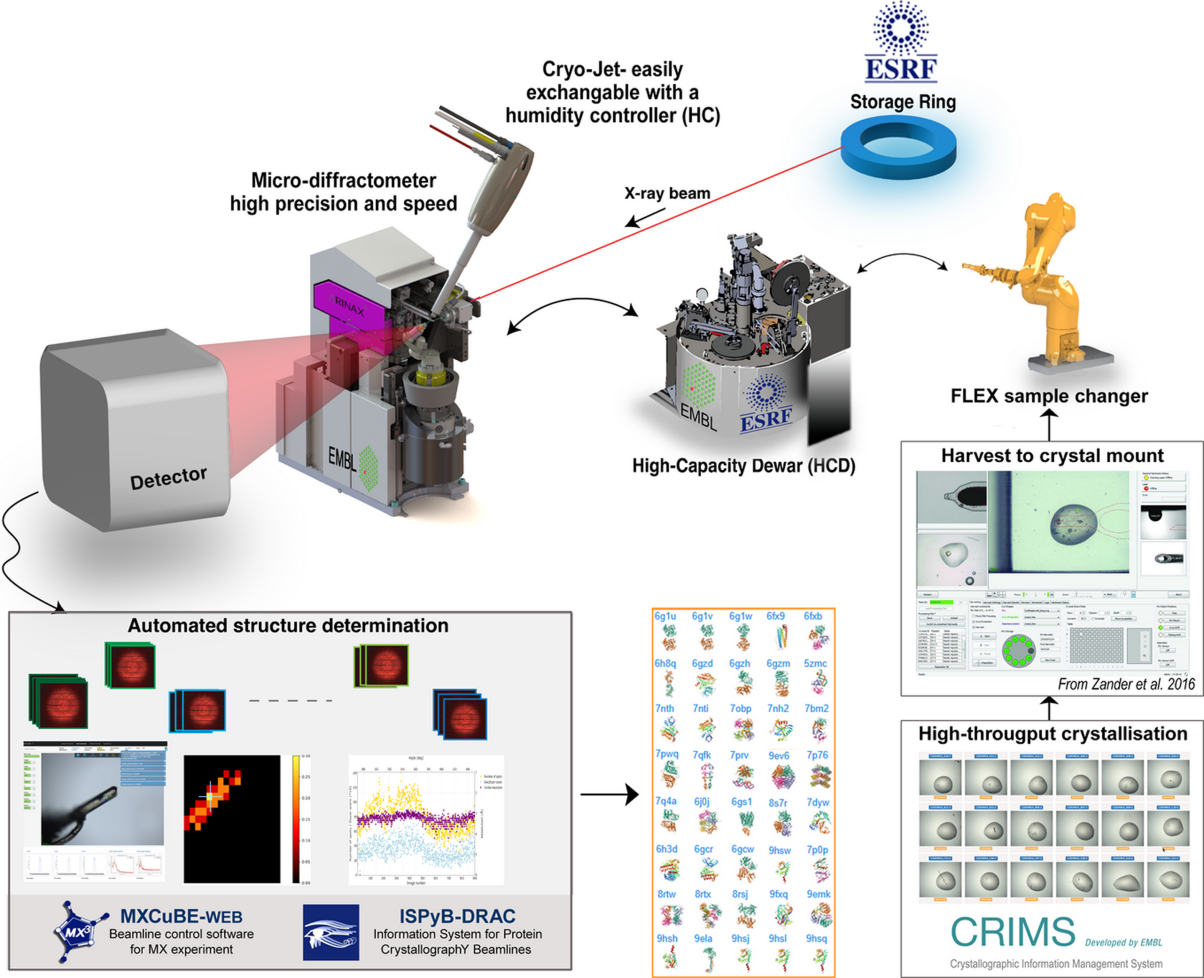
Automated MX pipeline for crystal mounting to structure envisaged at MASSIF-1 using the micro-diffractometer (MD2S), Flex sample changer with high-capacity dewar (FlexHCD) and automated crystal-harvesting robot, beginning with high-throughput crystallization available through CRIMS at the HTX platform at EMBL. Crystal characterization, data collection, data processing and structure determination are fully automatic using the *MXCuBE-Web* frontend and *ISPyB-DRAC* backend software tools. Structure gallery from MASSIF-1, retrieved and reproduced with permission from *BioSync* (https://biosync.rcsb.org/) (Kuller *et al.*, 2002 ▸).

Ongoing advances in robotic and detector technologies have significantly increased the efficiency of synchrotron beamlines, particularly in MX. The ESRF has led the way in developing software for controlling diffraction experiments, notably *MXCuBE*, which has since become part of a global collaboration adopted by many synchrotron facilities (Oscarsson *et al.*, 2019 ▸). Remote and fully automatic operation have become the standard for MX experiments, while a flexible interface is still essential to support automated data acquisition and tailor it to specific beamline requirements. The latest *MXCuBE* version (*MXCuBE-Web*) has been developed using web-technologies, enhancing remote access in a secure manner and allowing advanced experiments to be performed through any modern browser. In the near future, automation is expected to reach new levels with better integration of modern experimental database management systems and advanced data analysis tools, extending beyond traditional data processing to include complete structure determination and ligand identification (Fig. 3 ▸).

It is clear that the increased throughput of MX, SSX, SAXS and EM with data handling from the low level (moving and storing data) through to user presentation layers continues to be challenging. Many of the systems that served us very well previously cannot be scaled to current and future experiments. To handle this, numerous aspects of optimization are already under development. For example, hit finding for SSX and grid scans must occur early in the process, reducing the load on data storage, processing and backup. Initial work in this domain using LIMA2 on ID29 has already been shown to be robust and scalable (Kieffer *et al.*, 2025 ▸) and soon available on the other MX beamlines with EIGER2 and PILATUS4 detectors. Data processing via the use of GPUs and or FPGAs can also be envisioned for tasks such as hit finding and indexing. Processing requirements will of course also increase, but as long as I/O rates can continue to scale, the addition of more computer nodes still offers a path towards supporting peak loads. No less important is the careful cataloguing of experimental metadata and processing. To this end, the modernization and migration of our existing *ISPyB* experimental database to *ISPyB-DRAC* is underway to overcome limitations imposed by how previous MX experiments were performed. This migration will leverage maintainable software technologies that offer more agile responses to this fast-changing field. Finally, the prospect of moving some of these components to cloud computing providers must be carefully evaluated on a technical (cost effectiveness, data throughput and backup) and policy level (data confidentiality, data ownership).

On the other hand, artificial intelligence (AI) and machine-learning (ML) techniques, such as those powering *AF2*, heavily rely on structural datasets for training (Varadi *et al.*, 2024 ▸). Although exact lists of PDBs used for training *AF2* are unavailable, using the *OpenProteinSet* (Ahdritz *et al.*, 2023 ▸), we estimate that almost 85% of the structures used to train *AF2* originate from synchrotron datasets, emphasizing the critical role synchrotron MX has played in enabling this key technology. We know major classes of structures are underrepresented in the PDB, including room-temperature structures, chemically diverse ligand–protein complexes and time-resolved datasets. Therefore, significant work remains in order to facilitate the generation of new structural ‘ground truths’ for the further development of *AF2*-like advances. The adoption of advanced algorithms that incorporate the AI/ML developments must be foreseen on our X-ray beamlines and cryo-EM/ET microscopes to improve many aspects of our existing experimental and analyses workflows. For example, AI-based image recognition for sample centring, particle identification and hit finding; and ML methods that extend on current methods for treating non-isomorphism are envisioned. Indeed, many of the developments in automated workflows could be extended to include AI-based decision making (for example, based on mining the *ISPyB* database) that could be pushed ‘upstream’ into *MXCuBE-Web* for improved dynamic experimental strategies.

These advances in software are continually being implemented and developed across our beamlines to enhance experimental efficiency and precision. At MASSIF-1, innovations in sample handling, such as the integration of an upgraded crystal-harvesting robot (Felisaz *et al.*, 2019 ▸; Zander *et al.*, 2016 ▸; Cornaciu *et al.*, 2021 ▸) that enables automated near-physiological data collection, will drive large-scale room-temperature experiments in MX (Naschberger *et al.*, 2019 ▸). These developments allow for the preservation of biomolecular flexibility and dynamics, providing insights into conformational states often obscured in cryo-cooled samples (Skaist Mehlman *et al.*, 2023 ▸). Opening energetic and conformational landscapes is valuable in fragment- and compound-screening campaigns. This approach enables the collection of high-quality data, making it particularly advantageous for extensive fragment-based drug discovery (FBDD) campaigns. Similarly, ID23-1 is enhancing room-temperature FBDD with planned humidity-controlled storage systems to maintain sample integrity. The ability to collect SSX data on ID23-2 directly from crystallization plates ensures challenging radiation-sensitive drug targets can also be studied (Foos *et al.*, 2024 ▸). Together, this exemplifies the ESRF–EMBL JSBGs commitment to address the increasing demand for higher throughput and greater sample capacity, leveraging feedback from industrial users to achieve performance tailored to industry standards.

Furthermore, automation can also address a major problem in data collection: the ‘missing wedge’ of data that can occur with low-symmetry space groups. This can be alleviated by collecting multiple orientations and merging the sweeps, but this is a complex and time-consuming process that few users are willing or able to perform. If a missing wedge is predicted following characterization in our fully automated workflows on MASSIF-1, a multi-orientation strategy is now calculated. Once data collection in the first orientation is completed, the mini-kappa goniometer (Brockhauser *et al.*, 2013 ▸) fitted on the MD2S diffractometer is opened and a second round of X-ray centring triggered. The second sweep is then collected at the kappa angle that has been predicted by the workflow. This strategy allows for the acquisition of complementary diffraction data, which ensures better coverage of reciprocal space. Both datasets are then processed separately by the beamline’s automated data-processing pipeline. Afterwards, the two datasets are merged together to create a fully complete and accurate set of reflections, improving the overall quality of the structural information (Vonrhein *et al.*, 2011 ▸).

### 3.2. Future developments in serial crystallography

Over the past decade, the field of SSX has evolved significantly, leading to the development of diverse sample delivery approaches. Techniques, such as injector systems and fixed targets, have gained widespread adoption at synchrotron and XFEL facilities, encouraging discussions on standardization (Owen *et al.*, 2023 ▸), and are now complemented by hybrid methods, such as microfluidic devices and tape drive systems (Nam & Cho, 2021 ▸; Kamps *et al.*, 2024 ▸). Each delivery method offers distinct advantages such as sample efficiency, reaction initiation strategies or access to particular reaction time domains, making it essential for a user facility to provide diverse options to accommodate their requirements for TR-SSX. Sample delivery methods will continue to develop in the future, likely prioritizing automation, while aiming to provide cost-effective and sample efficient approaches, which in turn lowers the entry barriers for new users. This requires new automated data-processing routines to cope with the higher throughput of data. Additionally, TR-SSX greatly benefits from complementary spectroscopic techniques to disambiguate convoluted mixtures of chemical states often encountered under turnover conditions. In the future, advanced data analysis tools, supported by statistical models, could enhance characterization of intermediates or interpolate between recorded time points. Room-temperature data collection is more physiologically relevant for ligand-binding studies (Dunge *et al.*, 2024 ▸), which can differ from cryogenic observation, and is particularly useful to identify dynamics. High-pressure crystallography similarly complements TR-SSX by revealing intrinsic protein dynamics and enabling studies of interactions of gaseous molecules with enzymes.

**Figure 4:**
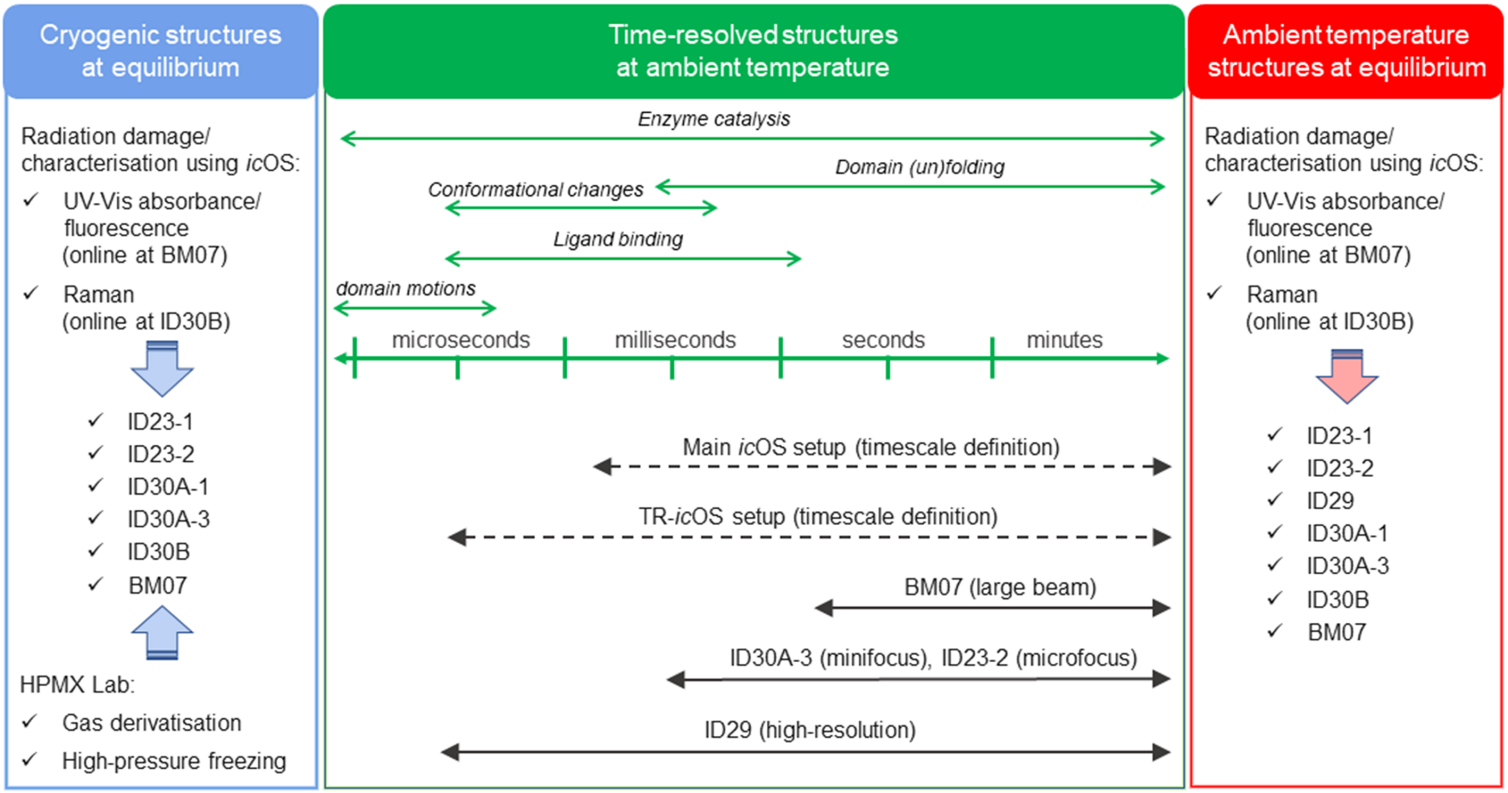
Synergic use of the ESRF MX beamlines and associated facilities in the favourable case when optical spectroscopic data can be accumulated on a crystalline protein containing a coloured cofactor, substrate or product. Crystallographic and spectroscopic data can be recorded at equilibrium at cryogenic (blue area) or ambient temperature (red area). Time-resolved (out of equilibrium) data recorded at room temperature are represented in green. Depending on the beamline or facility, crystals can be single ones (MX) or microcrystals (SSX).

At the ESRF, user demand in the Structural Biology group for SSX experiments is met by providing users with high-throughput beamlines such as ID23-2, MASSIF-3 and ID30B (Fig. 4 ▸). These are ‘standard’ MX beamlines, which can accommodate room-temperature and time-resolved experiments on slower time scales. Development of *in situ* serial crystallography (*i*SX) at ID23-2 allows for room-temperature data collection on batch grown crystals in 96-well plates (Foos *et al.*, 2024 ▸). This high-throughput approach reduces sample consumption (∼10 µg per dataset) and is ideally suited for exploration of novel biological targets, and may in the future see applications for room temperature ligand-binding studies. In addition, time-resolved oscillation crystallography (TR-SOX), developed at MASSIF-3, leverages data collected from crystal wedges to reduce sample consumption (∼3 µg per dataset) by decreasing the number of crystals per dataset (10 to 100 crystals) (Aumonier *et al.*, 2020 ▸). Installation of a fast chopper in combination with experiments based on the Hadamard approach (Yorke *et al.*, 2014 ▸) could see the time resolution of TR-SOX improve to the sub millisecond range. Meanwhile, ID29 is tailored to achieve higher TR-SSX from a wide range of sample delivery devices. Integration of software solutions for the ‘online’ and ‘offline’ processing of data are actively being developed and are important to further streamline the experimental decision-making process (Malla *et al.*, 2025 ▸; Orlans *et al.*, 2025 ▸). Moreover, a tuneable high-intensity nanosecond laser will soon be available for studies on photoactive proteins or with photocaged chemicals, and could potentially enable temperature-jump experiments. Additionally, these beamlines could see further integration with ‘offline’ or ‘online’ transient spectroscopic measurements provided by the *ic*OS Lab (Engilberge *et al.*, 2024 ▸). Expanding the available spectroscopic techniques, such as infrared (IR) absorption or X-ray spectroscopies, would enable a broader variety of enzyme systems to be studied and address some limitations concerning optical spectroscopies on microcrystals (Makita *et al.*, 2023 ▸). Furthermore, NMX advancements at LADI-III and DALI complement MX by enabling studies of enzyme structures and dynamics under near-physiological conditions. This combination provides unique insights into catalytic mechanisms and protein–drug interactions often inaccessible with MX alone (Gajdos *et al.*, 2022 ▸). Similarly, time-resolved X-ray solution scattering (TR-XSS) at ID09 captures low-resolution, global protein structural changes and intermediate states with high temporal resolution. Recent developments in user-friendly tools based on MD simulations and advanced triggering methods have made TR-XSS more accessible, establishing it as a critical technique for investigating protein dynamics and structural transitions in solution (Pounot *et al.*, 2023 ▸).

### 3.3. Future developments in high-energy X-raycrystallography and gas-derivation analyses

Another strategic objective is to maintain and enhance versatile experimental capabilities, offering state-of-the-art beamlines and complementary techniques to its international user community. It has previously been shown that MX data collection at higher energies with quantum efficient CdTe sensors has many benefits, mainly due to a significant reduction in their parallax error. This includes higher signal-to-noise ratios and better spot resolution, leading to higher quality data at atomic resolution (<1 Å) (Donath *et al.*, 2023 ▸) to facilitate detailed chemical analysis of enzymatic reactions. A major upgrade on ID30B therefore involves extending the energy range of the beamline from the current 6–20 keV to a maximum of 30 keV, supported by the integration of a newly acquired PILATUS4 XE CdTe detector, enabling fast data collection rates of up to 2000 Hz. Simultaneously, the beam size will become adjustable from the current ∼25 µm diameter to a range of 20–200 µm, providing optimal beam–crystal matching through automated workflows. To complement diffraction studies, ID30B aims to implement automated X-ray fluorescence (XRF) data acquisition, enabling the unambiguous identification of heavier elements (*e.g.* Ca, Fe, Zn, Cu) in crystals. Coupled with the existing Raman spectroscopy capabilities, this advancement will provide users with richer datasets. Furthermore, the proximity to the HPMX laboratory and its expanding inventory of gas-derivatization pressure cells (currently including Ar, Kr, Xe, O_2_, CO_2_, CH_4_) facilitates macromolecular studies with biologically relevant gases. Future developments will focus on expanding the range of gases, such as H_2_S and NH_3_, with pressure cells tailored to their thermophysical properties and phase diagrams. This will allow studies of gas–macromolecule interactions at ambient temperatures, crucial for light-driven macromolecules like rhodopsins. Additionally, the HPMX laboratory will advance investigations into enzyme–gas interactions and dynamic ligand binding in macromolecules (Prangé *et al.*, 2022 ▸), offering unprecedented insights into static and time-resolved crystallographic processes. These innovations underscore ID30B and the HPMX laboratory’s commitment to delivering cutting-edge tools for structural biology research.

### 3.4. Future perspectives in BioSAXS/bioSANS

At BM29, planned upgrades, including the integration of the SEU2B vacuum chamber, promise to significantly expand the capabilities of BioSAXS (Tully *et al.*, 2023 ▸). These enhancements pave the way for deploying state of the art microfluidic devices designed for high-resolution measurements of protein–ligand interactions and phase transitions, including liquid–liquid phase separation (LLPS). In collaboration with the EMBL through the JSBG partnership and the PSCM facility, notable innovations include a mixing chip capable of capturing real-time changes in protein–ligand, protein–salt and protein–pH interactions at millisecond timescales, as well as a gel-chip tailored for analysing highly viscous materials like hydro­gels. These tools will dramatically enhance the beamline’s ability to study protein condensates and LLPS *in situ*. Looking ahead, a move to *ISPyB-DRAC* and the upgrade of our processing pipeline is also under development, with the addition of *AF* predictions (Grudinin *et al.*, 2021 ▸), normal mode analysis tools (Laine & Grudinin, 2021 ▸) and the introduction of molecular dynamics calculations to model conformational flexible systems (Pelikan *et al.*, 2009 ▸). This will allow researchers to rapidly assess *AF* predictions directly from amino acid sequences (Brookes *et al.*, 2023 ▸). These advancements will address pivotal questions in cell biology, including protein dynamics, structural changes during ligand binding, the formation of protein condensates and hydro­gels, and the role of dynamic structures in disease progression and cellular regulation.

SANS at the ILL is also undergoing significant upgrades to enhance its capabilities for structural biology. The D11 instrument now features increased neutron flux and a new detector, extending its *Q*-range and dynamic range, while a refractive index (RI) detector has been integrated into the SEC–SANS setup. This addition enables more precise characterization of protein–detergent and protein–nanodisc complexes. On D22, a new autosampler capable of processing up to 96 samples is being commissioned, improving buffer subtraction quality and optimizing beam time efficiency. Future developments aim to harmonize biophysical platforms, deuteration facilities, BioSAXS and BioSANS, creating a comprehensive environment for biomolecular characterization (Mizuta *et al.*, 2018 ▸). Advances in match-out nanodiscs and natural lipid systems are expected to significantly enhance the study of membrane proteins in their native lipid contexts. Additionally, tools such as the *Pepsi-SANS* web app are being developed to integrate SANS data with structural prediction platforms like *AF* (Grudinin *et al.*, 2021 ▸). Though neutron flux remains a limiting factor for full automation, these upgrades are set to drive substantial progress in the structural biology techniques offered.

Furthermore, higher-resolution SAXS measurements over an extended *Q*-range can be achieved in collaboration with the ID02 beamline, which provides high brilliance and supports time-resolved SAXS, with temporal resolutions ranging from microseconds to milliseconds. These capabilities make ID02 ideal for capturing rapid dynamic processes and structural changes in biological systems, enabling the detection of even smaller changes. Such precision is particularly useful to model loop movements in macromolecules (Narayanan *et al.*, 2021 ▸; Makowski, 2010 ▸; Cho *et al.*, 2021 ▸), serving as an excellent complement to bioSAXS and SANS experiments.

Lastly, SAXS/SANS/WAXS demonstrates its strong interdisciplinary value when they are integrated with complementary techniques such as atomic force microscopy, biochemical analysis and models from MX, cryo-EM and NMR. Notably, the high-field NMR facility at the IBS provides atomic-scale resolution of intrinsically disordered regions (IDRs) and hydro­gels, offering insights that complement scattering-based findings (Hutin *et al.*, 2023 ▸).

### 3.5. Future developments in cryo-EM, cryo-ET and X-ray tomography

At CM01, the primary objective is to enhance throughput and automation by establishing seamless pipelines for SPA and cryo-ET. Key advancements include fringe-free imaging (FFI), which enables the use of smaller beams to cover a larger number of images within a given area of an EM grid, thereby significantly increasing throughput. FFI will be retrofitted into our G3 microscope. By 2027, the microscope’s camera and filter will undergo upgrades to incorporate state of the art technologies. Additionally, the real-time processing pipeline will be expanded to encompass 3D processing steps by employing several software packages to ensure convergence toward solutions. This will enable instant feedback during experiments, enhancing data quality and efficiency. By optimizing workflows to push resolution limits in CM01 and CM02 and incorporating AI-driven tools for particle picking and classification within *SCIPION* we can reduce user intervention and improve the overall data quality. These developments will accommodate increased SPA throughput while maximizing the efficiency of the Krios microscope operation. Taken together, these advancements will position the EPN campus as a leader in providing comprehensive, end-to-end cryo-EM/ET solutions, such as the SOS service pipeline (Fig. 2 ▸).

Though individual imaging methods provide detailed, stand-alone views of macromolecules or their functions, a multimodal imaging approach offers a more comprehensive understanding of complex biological processes. Cryo-EM stands out for achieving the highest resolution, with increasing integration of cryo-ET into multimodal approaches (Harkiolaki *et al.*, 2018 ▸). Recent innovations aim to enhance throughput and compatibility between volume electron microscopy (vEM) and cryo-ET (McCafferty *et al.*, 2024 ▸; Pierson *et al.*, 2024 ▸). However, throughput in cryo-ET faces challenges, particularly in lamella preparation, including target prediction and axial registration inaccuracies. While advances like focused ion beam scanning electron microscopy (FIB–SEM) and plasma FIB mitigate these issues to some extent, further improvements in lamella preparation techniques are essential. Enhancing precision in axial registration and developing more effective lamella preparation technologies are thus urgent priorities.

Using X-ray phase contrast imaging it is possible to study larger biological samples in 3D at cellular resolution. Using bigger samples, rare biological events can be located and targeted for high-resolution imaging using vEM techniques with significantly reduced acquisition times. The first X-ray tomography experiments to visualize protein crystals at an MX beamline were carried out on ID14-4 in 2008 (Brockhauser *et al.*, 2008 ▸). However, due to various difficulties this was not pursued any further at that time. In 2019, a similar experiment on P14 (EMBL Hamburg) at PETRA III was more successful (Polikarpov *et al.*, 2019 ▸). This went on to form the basis for a high-throughput tomography (HiTT) platform for phase-contrast imaging of biological samples that offers fast and versatile biological imaging capabilities (Albers *et al.*, 2024 ▸). This platform on the P14 high-photon-flux undulator beamline enables tomographic phase-contrast acquisition in about 2 min. The system is linked to an automated data-processing pipeline that delivers a 3D reconstructed dataset in under 90 s after completion of the X-ray scan. The integration of an MX robotic sample changer further enables the streamlined collection and reconstruction of X-ray imaging data from potentially hundreds of samples during a beam time shift. HiTT has been successfully applied to various biological tissue samples and paraffin-embedded preparations (Albers *et al.*, 2024 ▸). The ESRF–EBS upgrade, long length (>100 m from source to sample position) and a recent change to 2D Be CRLs for X-ray focusing make ID30B an ideal beamline to replicate this success. Initial feasibility studies were encouraging and, if successful, we plan to expand ID30B capabilities to offer soft biological sample imaging at cellular resolutions (<1 µm). By capitalizing on the well established MX infrastructure such as automated sample changers, reliable beamline instrumentation and user-friendly interfaces, we aim to achieve rapid data acquisition and reconstruction, with 3D volumes available within minutes. This streamlined process will make X-ray micrometre resolution imaging accessible to structural biologists, bridging the gap between structural biology and cellular/tissue imaging. We also plan to implement it as a screening facility, complementing existing X-ray imaging beamlines at the ESRF such as the nano-probe imaging beamline on ID16A. We hope to offer HiTT to external users for the rapid collection of tomography data from diverse samples and energies in the not too distant future.

## 4. Conclusions

With more than 18000 PDB structures and 16000 publications stemming from structural biology experiments carried out at ESRF and ILL, the Grenoble EPN campus is well positioned to lead the structural biology community into a new era of integrated, multiscale and multimodal research. By advancing cutting-edge technologies such as high-throughput cryogenic and room-temperature MX, TR-SSX and SAXS/SANS the campus can address key challenges in understanding macromolecular dynamics. Developments in AI-driven workflows, data processing and complementary spectroscopic techniques will further enhance experimental efficiency and accuracy. Innovations in cryo-EM, cryo-ET and X-ray tomography will bridge the gap between molecular and cellular scales, offering transformative insights into biological processes. With strategic upgrades across beamlines and instruments, including SAXS, SANS and high-energy X-ray analysis, the campus is committed to providing unparalleled research infrastructures for dynamic, time-resolved and physiologically relevant studies. Together, these advancements will solidify the EPN campus’s role as a hub for interdisciplinary research, driving breakthroughs with far-reaching impacts in the life sciences.

## Figures and Tables

**Table 1:** Overview of the key characteristics of the MX beamlines and EM facilities at the ESRF that are dedicated to structural biology

Straight section	BM07	ID23		ID29	BM29	ID30				
Beamline	BM07 (FIP2)	ID23-1	ID23-2	ID29	BM29	ID30A-1 (MASSIF-1)	ID30A-3 (MASSIF-3)	ID30B	CM01	CM02
Method	MX†	MX†	MX‡	SMX	SAXS	MX§	MX¶	MX†	Cryo-EM/ET	Cryo-EM/ET

X-ray characteristics									Electron energy	
Energy range (keV)	6–20	6–20	14.2	10–25, 35	7–15	12.8	12.81	6–20	300 kV	300 kV
Flux (photons s^−1^)	6 × 10^11^	4 × 10^12^	>10^13^	2 × 10^15^	1 × 10^13^	2 × 10^12^	1 × 10^13^	>10^13^	N/A	N/A
Beam size, H × V (µm)	200 × 100	10 × 10	1.5 × 3	0.5 × 0.5	0.2 × 0.1	10 × 10	15 × 15	20 × 30	N/A	N/A
	250 × 250	45 × 30	8 × 25	6 × 5		100 × 100		200 × 200		

Large beamline instrumentation										
Diffractometer	MD2M	MD2S	MD3Up	MD3UpSSX	–	MD2S	MD2	MD2S	N/A	N/A
Sample changer	FlexHCD	FlexHCD	FlexHCD	FlexHCD	BioSAXS SC	FlexHCD	FlexHCD	FlexHCD		
Detector (sensor)	PILATUS2 6M	EIGER2 16M (CdTe)	EIGER2 X 9M (0.45 mm Si)	JUNGFRAU 4M	PILATUS3 2M (0.45 mm Si)	PILATUS3 6M (1 mm Si)	EIGER1 X 4M	EIGER2 X 9M (0.45 mm Si)	Gatan K3	Falcon 4i
						From 2026, PILATUS4 4M (Si)		From 2026, PILATUS4 XE 4M (CdTe)		
Additional	KETEK XRF detector	KETEK XRF detector	Mini-kappa goniometer	Millisecond laser	SEC-SAXS	Mini-kappa goniometer		KETEK XRF detector		
	Mini-kappa goniometer		*In situ* plate holder	Nanosecond laser	SEU2B with microfluidics	*In situ* plate holder		Mini-kappa goniometer		
	Online microspec							*In situ* plate holder		
Operating since	2021	2004	2005	2022	2014	2014	2014	2014	2017	2024

† Tuneable.

‡ Microfocus.

§ Automated.

¶ Minibeam.

**Table 2:** Overview of the key characteristics of the ILL instruments involved in structural biology

Guide	Cold neutron guide H15	Cold neutron guide H512		Cold guide H143	Cold guide H141
Instrument	D11	D22		LADI-III	DALI
Experimental method	SANS	SANS		NMX	NMX

Monochromator					
	Velocity selectors			Quasi-Laue Ni/Ti multilayer band-pass filter (δλ/λ = 30%)	Quasi-Laue Neutron velocity selector (δλ/λ = 10%)
	Wavelength 4.5 < λ (Å) < 40			λ range 2.8 to 3.8 Å	λ range 3.35 to 3.70 Å
	Wavelength spread 10% (20% by tilting the selector)			λ centre = 3.3 Å	λ centre = 3.53 Å

Collimation					
Segments	1.6, 3.1, 4.1, 5.6, 6.7, 7.7, 10.2, 12.7, 15.2, 16.5, 17.8, 20.3, 22.8, 24.8, 26.9, 29.4, 31.9, 35 m	2.0, 2.8, 4.0, 5.6, 8.0, 11.2, 14.4, 17.6 m		Pinholes	Pinholes
Guide section	45 mm × 45 mm, with the first 3 segments elliptical	40 mm × 55 mm		0.5 to 2.9 mm	0.5 to 3.0 mm

Sample area					
Maximum flux at sample position	8 × 10^7^ n cm-2 s^−^ for λ = 6 Å ± 10%	1.2 × 10^8^ n cm-2 s^−1^ for λ = 6 Å ± 10%		∼1.1 × 10^8^ n cm^−2^ s^−1^ (λ centre = 3.3 Å; δλ/λ = 30%)	∼1 × 10^8^ n cm^−2^ s^−1^ (λ centre = 3.53 Å; δλ/λ = 20%)
Sample size	10–300 mm^2^	10–300 mm^2^			

Sample environment					
	22-position rack, rectangular Hellma cuvettes (180 µl)			Ambient or Oxford Cryosystems ‘COBRA’ cryostream (down to 80 K)	
	15-position rack, banjo Hellma cuvettes (300 µl)				
	High pressure cell 3 kbar	Autosampler (96 samples/ 200 µl); SEC–SANS; 4-position rack, dialysis cells			

Detector					
	^3^He tube detector	^3^He tube detector		Neutron image plate Gd2O3-doped BaF(Br.I):Eu2+	Neutron image plate Gd2O3 doped BaF(Br.I):Eu2+
		**1**	**2**		
Area	2 m^2^	1 m^2^	0.6 m^2^	1250 mm × 450 mm	1000 mm × 400 mm
Distance	1.4–38 m	4–17.6 m	1.4 m		
Rotation	0°	0°	20°		
*Q*-range	0.0003–0.8 Å^−1^	0.001–1 Å^−1^			
Maximumcount rate	5 MHz	5 MHz			
Pixel size	8 mm × 4 mm	8 mm × 4 mm		125, 250, 500 µm^2^	100, 200, 400 µm^2^
Radius				200 mm	160 mm
Length				450 mm	400 mm

Operating since	1972	1995		2012	2024
